# Recurrent Blister Formation in Setting of Poorly Managed Diabetes Mellitus

**DOI:** 10.7759/cureus.5029

**Published:** 2019-06-28

**Authors:** Michael J Willcox

**Affiliations:** 1 Medicine, Tulane University School of Medicine, New Orleans, USA

**Keywords:** bullae, diabetes, bullous diabeticorum

## Abstract

Bullous diabeticorum is a condition of unknown etiology with abrupt blister formation and spontaneous resolution. While commonly thought as rare, it is likely underdiagnosed resulting in mismanaged care and increased morbidity for individual patients. A shift in focus from empiric treatment to appropriate diagnostic workup is critical for this condition in the diabetic population.

## Introduction

Cutaneous conditions are just some of the numerous morbidities that impact patients with diabetes mellitus [1]. Many of these potential complications have similar initial appearances but varying pathomechanisms. The treatments for the conditions can at times be contradictory, thus demonstrating the importance of accurate workup [1-3]. Awareness of the various cutaneous manifestations, the appropriate workups or treatments, and the importance of managing the correlated poor glycemic control are important for any general practitioner caring for this population [4].

## Case presentation

A 66-year-old Caucasian man presented to the outpatient internal medicine clinic with concern for a non-healing ulceration on his left fourth toe and a large, previously ruptured blister on his left heel.

The patient had previously undergone numerous detachments and amputations of digits on all four extremities, including a below knee amputation of his right leg one year prior. The patient reported that all of these surgeries stemmed from spontaneous blisters on his digits. Most of the blisters would ultimately rupture, with many becoming non-healing ulcers despite aggressive antibiotic therapy and debridements.

The patient had previously undergone dermatologic workup for the frequent blistering. Histological samples were inconclusive and negative on immunofluorescent staining.

The patient’s past medical history includes hypertension, hyperlipidemia, peripheral vascular disease, and type II diabetes mellitus. The patient’s diabetes had been poorly controlled for many years, with hemoglobin A1c values averaging over 10 for the last decade. During this time, the patient developed sequelae of diabetic neuropathy, repeated diabetic foot ulcers with cellulitis, and end-stage renal disease with recently initiated peritoneal dialysis. Following the initiation of peritoneal dialysis, the patient’s glucose levels have maintained a normal range.

**Figure 1:**
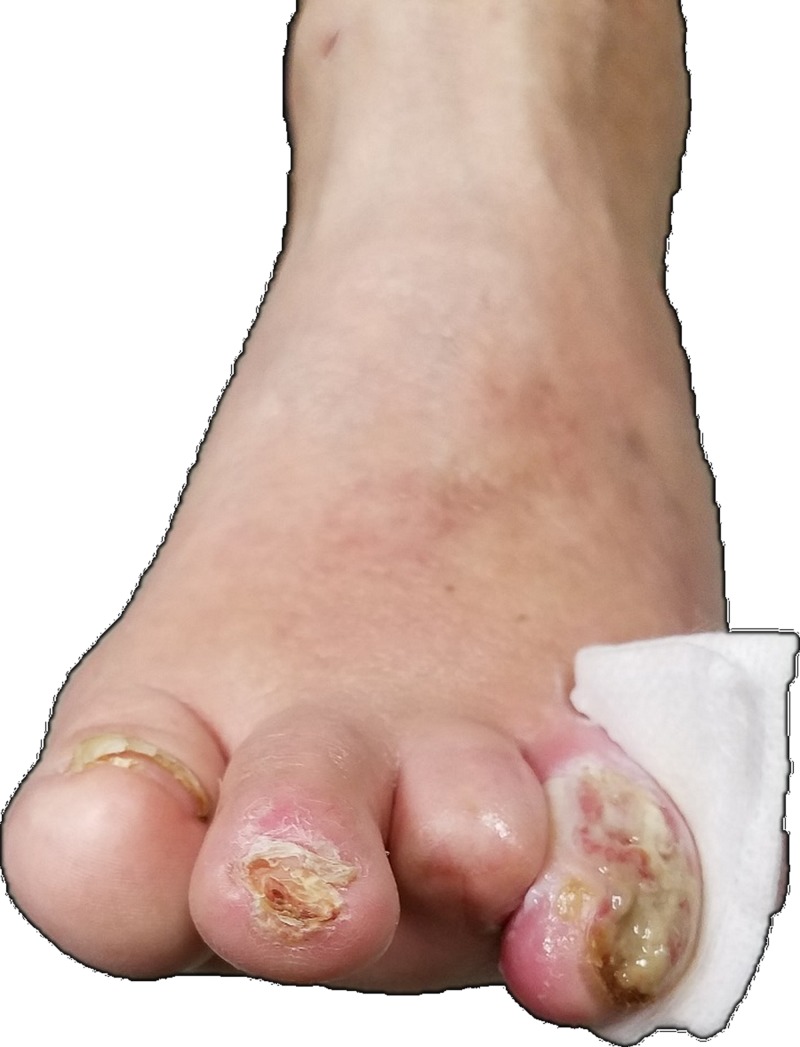
Left foot with ulceration and exudate from the fourth toe. Also visible is previously amputated third toe.

Physical examination reveals ulceration with purulent exudate of the left fourth toe from the entire nail bed continuing dorsally and medially to the mid-proximal phalanx (Figure 1).

**Figure 2:**
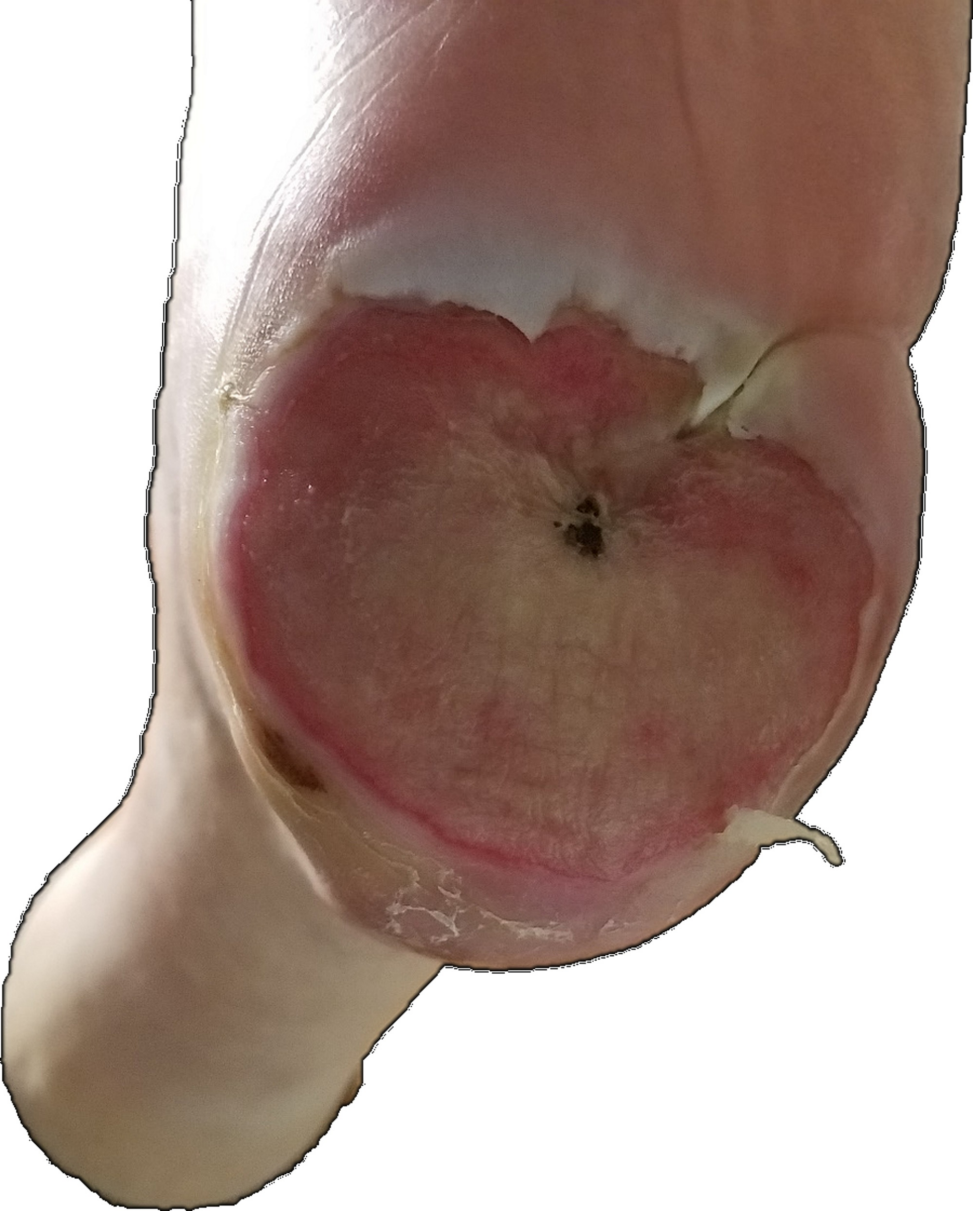
Left heel with large lesion from previously ruptured blister. Surface is dry and firm.

Exploration of the wound revealed tracking with bone involvement and suspected osteomyelitis. The left plantar surface has a large lesion spanning the entire width of the heel with apparent reepithelization and a small eschar appearance in the center (Figure 2).

**Figure 3:**
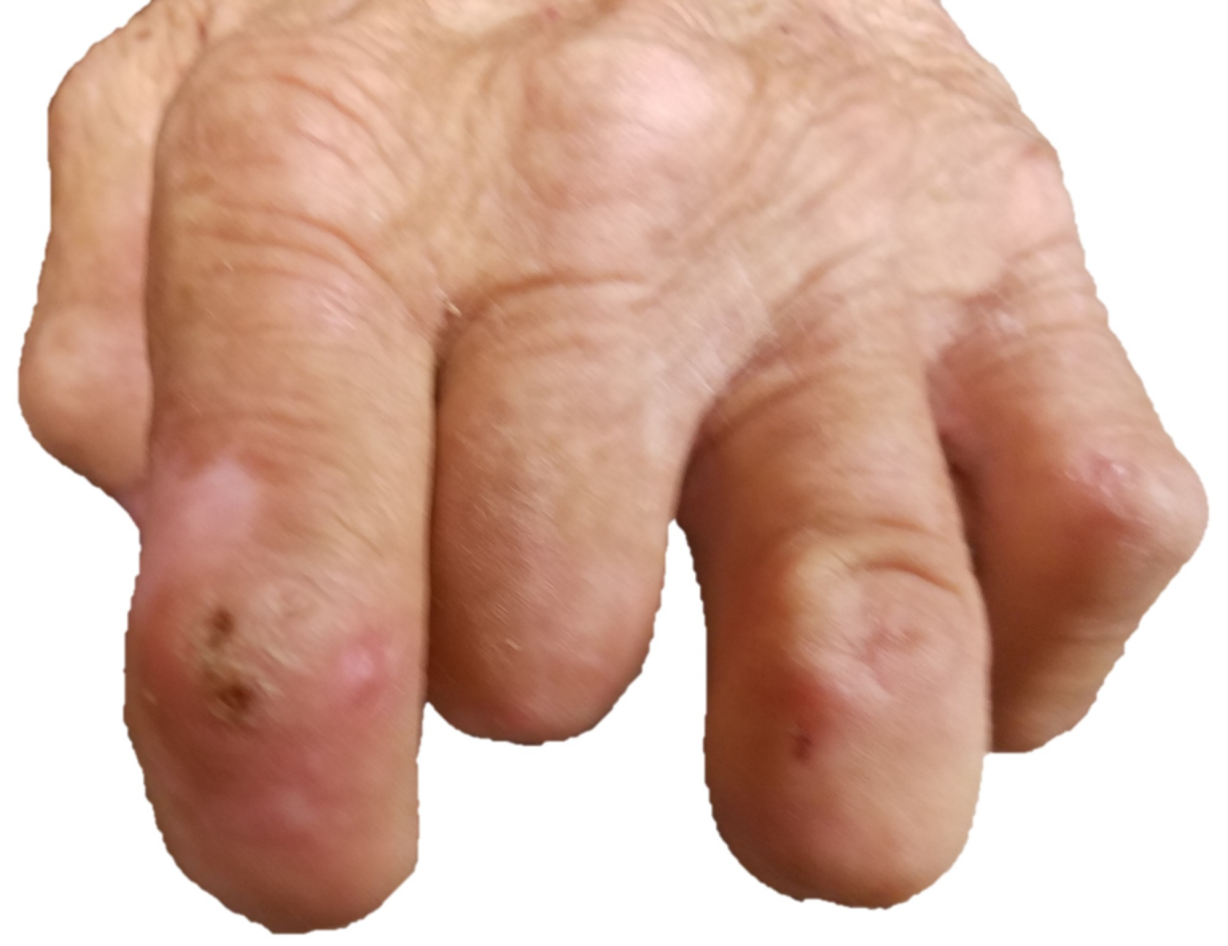
Left hand with well healed amputations of index, middle, and ring fingers.

**Figure 4:**
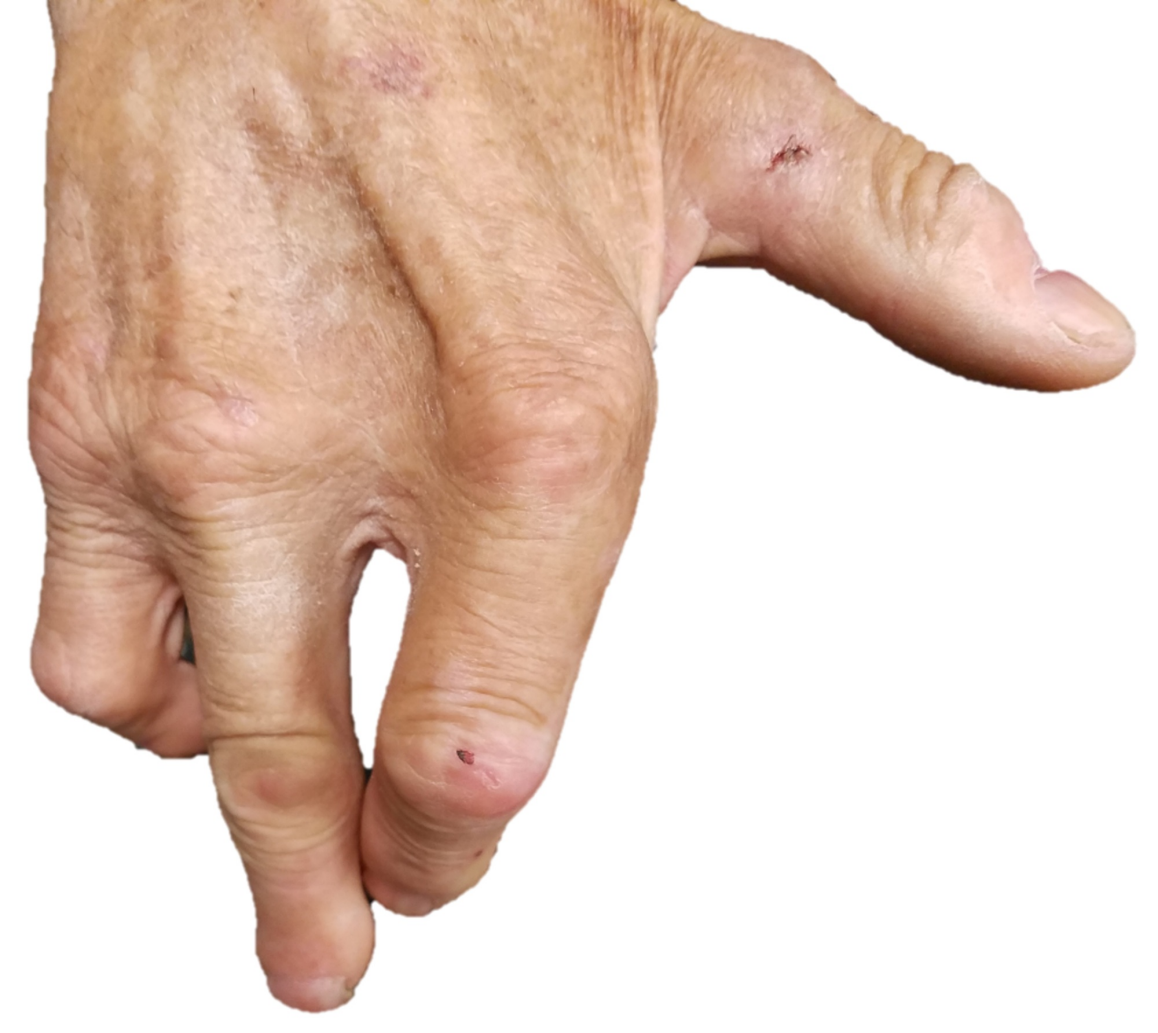
Right hand demonstrating ray amputation of the middle finger.

The area is red and dry, with the appearance of a previously ruptured large blister. The rest of his foot and both hands revealed numerous previous detachments and amputations, but no present ulcerations or blisters (Figure 3-4).

The patient was referred to the wound care team for recommendations on debridement with antibiotics vs surgical management of left fourth toe, as well as recommendations for aggressive management of recently ruptured plantar blister.

## Discussion

Cutaneous manifestations of diabetes are present in 30-70% of patients [1-4]. Bullous eruptions are not as common as bacterial and fungal infections, but the incidence is high enough to warrant a periodic review of the varied causes [3,5].

Bullous diabeticorum, also known as bullosis diabeticorum, presents as spontaneous eruptions of tense, serous, and sterile fluid-filled blisters on otherwise normal skin [6-11] of patients suffering from diabetes mellitus. They appear most frequently in the acral region: with the feet [6,8,9,12,13], and at times hands [3,5], being the most common. The blisters appear abruptly [12], without history of trauma [5], pain, signs of inflammation [3,11], or any further symptoms [12]. Reported blisters have a significant variance in size, from 0.5 to 10 cm [4,10]. They tend to have a self-limited evolution, with reepithelization of the blister floor occurring rapidly within a few days [12] and most resolving in 2-6 weeks without scarring [2,4,5,8,9,11,13,14]. However, most cases will reoccur [3,7,8,9,13,14]. The actual incidence of bullous diabeticorum remains uncertain, with studies presenting an annual incidence in patients with diabetes of 0.16-0.5% (1 in 200 to 625 patients) [4,15,16].

At the present time, the etiology remains unclear [2,3,4,8,14]. The most common presenting comorbidities are diabetic peripheral neuropathy [3,8], followed by vasculopathy [3,5,10]. Other less commonly presenting comorbidities include nephropathy [13,17], microangiopathy [4,18], or disorders of metabolism of calcium, magnesium, and carbohydrates [2,5,14,19]. One observation is that, in cases that were properly documented, the patients appear to have poor glucose control at the time of the blisters' eruptions [4,5,11,15]. This was the case with the presented patient, with no new blister formation noted in the year following initiation of peritoneal dialysis and improved blood glucose regulation. Nonetheless, no presenting condition or comorbidity has been documented as present in all cases [3,5,10,12], causing speculation that the disease is multifactorial [11].

No specific laboratory test exists for the diagnosis of bullous diabeticorum [8,11,13], leaving it as a clinical diagnosis of exclusion [4,8]. It is important to exclude other known bullous conditions [8]; however, due to the increased risk of infection in diabetic patients, particularly in the lower extremities, biopsies should only be performed in recurrent cases [4,15]. Biopsies should be taken from the bubble region for histological analysis and from the perilesional zone for immunofluorescence evaluation [8,13]. Additional tests should not be necessary [8].

Histopathologically bullous diabeticorum is noted for its heterogeneity [18], leaving exams inconclusive. Findings may range from subepidermal blisters with sparse perivascular infiltrate to intraepidermal with surrounding spongiosis [4,10]. Both direct and indirect immunofluorescence will be negative [8,14], excluding conditions like bullous pemphigoid that would otherwise appear similar clinically and histologically [2,8,11].

With the treatment, course, and prognosis being so different from other immune, ischemic, or neuropathic lesions of diabetic patients [5], it is critical to perform an adequate workup. For example, a misdiagnosis of bullous pemphigoid or pemphigus would result in treatment with high dose corticosteroids, likely increasing risks and exacerbating the underlying diabetes mellitus [2].

Current treatment is supportive, with primary goals to reduce discomfort and minimize the risk of secondary infection [2,8,10,12]. Conventional therapy involves keeping the blister intact to cover the lesion and prevent secondary infection [8,13]. Some have chosen to lance intact bullae for controlled drainage, keeping the roof intact [4,11]. Opening the bullae would necessitate topical antimicrobials. Authors have noted that reoccurrence at these sites is common, but no statistics are available to compare their commonality with the recurrence of preserved bullae. With either method, patients should be encouraged to keep their lesion clean and protected [8,11] with cold compresses providing relief for some [2]. This would also be a valuable time to assess and emphasize proper blood glucose regulation [4].

## Conclusions

While bullous diabeticorum is generally considered to be a rare condition, the lack of a specific diagnostic test may cause it to be significantly underdiagnosed and underreported. The condition is likely misdiagnosed for other bullous disorders and treated inappropriately, causing an increase in already significant morbidity and the formation of chronic ulcers. Thus, practitioners should have an increased sensitivity for it and seek adequate workup rather than beginning empiric treatment. In addition to ensuring proper management of individual patients, utilization of consulting services in workup and reporting of discovered cases will result in better assessment of incidence and pathogenesis. With more information, hopefully, the pathogenetic mechanism underlying bullous diabeticorum can be established so that a definitive diagnostic method can be developed and therapy can be directed at treatment instead of simply symptom and sequela relief.

## References

[1] Braverman IM (1971). Cutaneous manifestations of diabetes mellitus. Med Clin North Am.

[2] Paltzik RL (1980). Bullous eruption of diabetes mellitus. bullosis diabeticorum. Arch Dermatol.

[3] Collet JT, Toonstra J (1985). Bullosis diabeticorum: a case with lesions restricted to the hands. Diabetes Care.

[4] Wilson TC, Snyder RJ, Southerland CC (2012). Bullosis diabeticorum: is there a correlation between hyperglycemia and this symptomatology?. Wounds.

[5] Allen GE, Hadden DR (1970). Bullous lesions of the skin in diabetes (bullosis diabeticorum). Br J Dermatol.

[6] Oursler JR, Goldblum OM (1991). Blistering eruption in a diabetic bullosis diabeticorum. Arch Dermatol.

[7] Mendes AL, Miot HA, Haddad Jr V (2017). Diabetes mellitus and the skin. An Bras Dermatol.

[8] Mota AN, Nery NS, Barcaui CB (2013). Case for diagnosis: bullosis diabeticorum. An Bras Dermatol.

[9] Kurdi AT (2013). Bullosis diabeticorum. Lancet.

[10] Hurley MY, Mattox AR (2013). Diagnosis and management of bullous disease. Clin Geriatr Med.

[11] Chatterjee D, Radotra A, Radotra BD, Handa S (2017). Bullous diabeticorum: a rare blistering manifestation of diabetes. Indian Dermatol Online J.

[12] Toonstra J (1985). Bullosis diabeticorum: report of a case with a review of the literature. J Am Acad Dermatol.

[13] Lipsky BA, Baker PD, Ahroni JH (2000). Diabetic bullae: 12 cases of a purportedly rare cutaneous disorder. Int J Dermatol.

[14] Bernstein JE, Medenica M, Soltani K, Griem SF (1979). Bullous eruption of diabetes mellitus. Arch Dermatol.

[15] Larsen K, Jensen T, Karlsmark T, Holstein PE (2008). Incidence of bullosis diabeticorum: a controversial cause of chronic foot ulceration. Int Wound J.

[16] El Fekih N, Zeglaoui F, Sioud A (2009). Bullosis diabeticorum: report of ten cases. Tunis Med.

[17] Cantwell AR, Jr. Jr., Martz W (1967). Idiopathic bullae in diabetics: bullosis diabeticorum. Arch Dermatol.

[18] Goodfield MJ, Millard LG, Harvey L, Jeffcoate WJ (1986). Bullosis diabeticorum. J Am Acad Dermatol.

[19] Kurwa A, Roberts P, Whitehead R (1971). Concurrence of bullous and atrophic skin lesions in diabetes mellitus. Arch Dermatol.

